# Lack of Curricular Responsiveness to Regional Musculoskeletal Disease Burden in U.S. Medical Education

**DOI:** 10.2106/JBJS.OA.26.00018

**Published:** 2026-10-15

**Authors:** Erica Olfson, Lachlan Anderson, Carl Berghult, Nathaniel Bridge, David Shau

**Affiliations:** 1 The Anne Marion Burnett School of Medicine at Texas Christian University, Fort Worth, Texas; 2 Texas Hip and Knee Foundation, Fort Worth, Texas; 3 John Peter Smith Hospital, Department of Orthopaedic Surgery, Fort Worth, Texas

## Abstract

**Introduction:**

Musculoskeletal (MSK) disorders are among the leading causes of disability worldwide and account for a substantial proportion of U.S. healthcare visits. Despite this burden, undergraduate medical education devotes limited time to MSK instruction. This study evaluates whether medical school priorities or regional MSK disease burden influence MSK curriculum duration.

**Methods:**

Publicly available data from 157 U.S. allopathic medical schools were reviewed; 78 schools with identifiable MSK curriculum duration were included. Schools were categorized by primary care emphasis and geographic region using Association of American Medical Colleges classifications. Regional MSK disease burden was obtained from the Burden of Musculoskeletal Diseases in the United States (BMUS) database. Descriptive statistics, independent t-tests, chi-squared testing, and analysis of variance (ANOVA) were used.

**Results:**

Among the 78 U.S. allopathic medical schools with available curriculum data, the mean MSK curriculum duration was 5.97 weeks (SD 2.72; median 6; range 2-14). Thirty-nine schools (50%) emphasized primary care. MSK curriculum duration did not differ between schools with and without a primary care emphasis (5.79 vs. 6.15 weeks, p = 0.561). No significant differences were observed across geographic regions using either the 4-region (p = 0.500) or 9-division (p = 0.920) classifications. Despite substantial regional variation in MSK disease burden, MSK curriculum duration remained relatively uniform.

**Conclusion:**

MSK curriculum duration was similar across medical schools, averaging around 6 weeks, regardless of institutional mission or regional disease burden. This suggests that curriculum duration does not vary according to these institutional characteristics and may help inform future discussions regarding undergraduate musculoskeletal curriculum design.

## Introduction

Musculoskeletal (MSK) disorders are among the leading causes of disability worldwide and contribute substantially to healthcare utilization, economic burden, and impairment in quality of life. In the United States, MSK conditions account for more than 20% of healthcare visits and represent up to 40% to 60% of encounters in primary care settings^1-4^. The prevalence and disability-adjusted life years associated with MSK disease also vary significantly across geographic regions and sociodemographic populations^1-4^. Given the frequency with which MSK conditions present in clinical practice, physicians across multiple specialties, particularly those in primary care, must possess the diagnostic and management skills necessary to appropriately evaluate these conditions.

Although previous studies have suggested that musculoskeletal education occupies a relatively small proportion of undergraduate medical curricula, objective comparisons across medical specialties remain limited. Most U.S. medical schools allocate less than 3% of total curricular hours to MSK topics, and relatively few institutions require a dedicated MSK or orthopaedic clerkship^1,5-12^. While this gap has been well documented, less attention has been given to whether MSK curricula reflect the healthcare needs of the populations that medical schools ultimately serve. Surveys in the United States and Canada indicate that schools serving regions with the highest prevalence of MSK conditions often provide some of the shortest formal MSK education, reflecting a persistent misalignment between local epidemiology and curriculum priorities^5,13^.

These trends underscore the need to evaluate how medical school mission (such as primary care emphasis) and regional MSK disease burden collectively shape the duration and structure of MSK curricula. This study evaluates whether musculoskeletal curriculum duration in U.S. allopathic medical schools varies according to institutional mission (primary care emphasis) or regional musculoskeletal disease burden. By examining these relationships, this study evaluates whether musculoskeletal curriculum duration varies according to institutional mission and regional musculoskeletal disease burden. These findings may help inform future discussions regarding factors associated with undergraduate musculoskeletal curriculum design.

This question is particularly relevant for institutions that emphasize primary care training. Because musculoskeletal complaints constitute a substantial proportion of visits in family medicine and primary care settings, one might expect institutions with a primary care mission to devote greater curricular emphasis to musculoskeletal education. Understanding whether musculoskeletal curriculum duration differs among institutions with varying educational missions may provide insight into 1 factor associated with undergraduate curriculum design.

## Materials and Methods

### Duration of MSK Curriculum

Publicly available curriculum data were reviewed independently for 157 U.S. allopathic medical schools by 2 authors with discrepancies resolved by a third author. Institutional websites, course catalogs, and published syllabi were examined. Total duration of dedicated MSK instruction was recorded in weeks; reported hours were converted using a standardized 40-hour instructional week. Schools without identifiable MSK-specific curriculum data were excluded.

### Presence of Primary Care Emphasis

Schools were classified as primary care–focused if their mission statements or program descriptions explicitly emphasized primary care or included a formal primary care pathway. Classification was performed independently by 2 reviewers with consensus resolution.

### Geographic Region Determination

Schools were categorized using both 4-region (Western, Central, Southern, and Northeastern) and 9-division AAMC geographic classifications (Pacific, Mountain, West North Central, East North Central, West South Central, East South Central, Northeast, Mid-Atlantic, and South Atlantic) based on their listed address.

### Musculoskeletal Disease Burden

Regional MSK disease burden data were obtained from BMUS, in September 2025, maintained by the U.S. Bone & Joint Initiative^14^. Adult prevalence, healthcare utilization, and activity-limitation metrics were extracted and linked to school location at the regional or state level. The Burden of Musculoskeletal Diseases in the United States (BMUS) database provides population-level estimates of musculoskeletal disease prevalence and healthcare utilization across U.S. regions. For this study, 3 indicators were selected as representative measures of MSK disease burden: (a) ambulatory MSK visit frequency, representing outpatient healthcare utilization for musculoskeletal conditions; (b) osteoporotic fracture–related visit frequency, reflecting regional burden of fragility fractures; and (c) acute musculoskeletal injury frequency, capturing traumatic and sports-related MSK presentations. These indicators were selected because they represent common MSK conditions encountered in both primary care and orthopaedic practice.

### Statistical Analysis

Descriptive statistics summarized MSK curriculum duration. Independent t-tests compared schools with and without primary care emphasis. ANOVA assessed differences across geographic regions. Statistical significance was defined as p < 0.05. Analyses were conducted using R (R Foundation for Statistical Computing, Vienna, Austria)^12^.

## Results

### Overview of Included Schools

Of the 157 U.S. allopathic medical schools examined, 78 had publicly available MSK curriculum information that met inclusion criteria. These schools formed the basis for all analyses. Their characteristics are summarized in Table 1. A total of 39 schools (50%) reported an explicit primary-care emphasis. Schools were distributed across all major AAMC regions, with the South containing the largest proportion (29 schools, 37%), followed by the Midwest/Central (20 schools, 26%), Northeast (17 schools, 22%), and West (12 schools, 15%).

**Table 1:** Characteristics of Included Medical Schools

Characteristic	N (%)
Total schools with MSK data	78 (49)
Primary care emphasis: yes	39 (50)
Primary care emphasis: no	39 (50)
Region-Northeast	17 (22)
Region-Midwest/Central	20 (26)
Region-South	29 (37)
Region-West	12 (15)

MSK = musculoskeletal.

### Association with Primary Care Emphasis

Because MSK disorders comprise a large proportion of primary care visits in the United States, we evaluated whether medical schools that emphasize primary care training provide greater exposure to MSK content. Despite this association, MSK curriculum duration was not significantly different between schools that identified a primary care emphasis and those that did not. Specifically, schools with a primary-care focus provided an average of 5.79 ± 2.63 weeks of MSK curriculum, whereas schools without this emphasis provided an average of 6.15 ± 2.80 weeks (p = 0.561), as given in Table 2.

**Table 2:** Institutional Characteristics and MSK Curriculum Duration

Institutional Factor	Mean Weeks ± SD	Median	p
Primary care emphasis (yes)	5.79 ± 2.63	5	p = 0.561
Primary care emphasis (no)	6.15 ± 2.80	6	—
Northeast region	**5.35 ± 1.90**	**5**	p = 0.500 (ANOVA)
Midwest/Central region	**6.60 ± 3.10**	**6**	—
South region	**6.10 ± 3.11**	**6**	—
West region	**5.50 ± 1.73**	**6**	—

Bolded entries represent regions as defined by the Association of American Medical Colleges. MSK = musculoskeletal.

### Geographical Variability in MSK Curriculum Duration

MSK curriculum duration was examined to determine whether it varied according to the 4 major AAMC geographic regions. As summarized in Table 2, schools in the Midwest/Central region demonstrated the longest mean MSK curriculum at 6.60 ± 3.10 weeks, followed by the South at 6.10 ± 3.11 weeks, the West at 5.50 ± 1.73 weeks, and the Northeast at 5.35 ± 1.90 weeks. These differences were not statistically significant (p = 0.500). Similarly, a more granular analysis using the 9-division AAMC classification also did not reveal statistically significant regional variation (p = 0.920), with length of exposure ranging between approximately 2 and 14 weeks.

### Lack of Alignment Between MSK Disease Burden and Curriculum Duration

To determine whether MSK curricula respond to regional population health needs, we compared curriculum duration with 3 BMUS-derived indicators of disease burden: ambulatory MSK visit frequency, osteoporotic fracture related visits, and acute MSK injury frequency (Figs. 1–3). Across all analyses, MSK curriculum duration showed minimal regional variation despite substantial differences in disease burden. As shown in Fig. 1, regions with the highest outpatient MSK visit volumes, including the Midwest and South, did not demonstrate longer MSK curricula compared with regions with lower visit rates. Instead, mean curriculum duration remained tightly clustered around approximately 5 to 6 weeks across all regions. Similarly, in Fig. 2, osteoporotic fracture–related visits were most frequent in the Midwest and South; however, MSK curriculum exposure in these regions was comparable with that of the Northeast and West, where fracture-related utilization was lower. In Fig. 3, acute MSK injury rates were consistently high across all regions, yet MSK curriculum duration again demonstrated little variability. Collectively, these findings suggest that musculoskeletal curriculum duration in U.S. medical schools appears largely independent of regional disease burden.

**Fig. 1:**
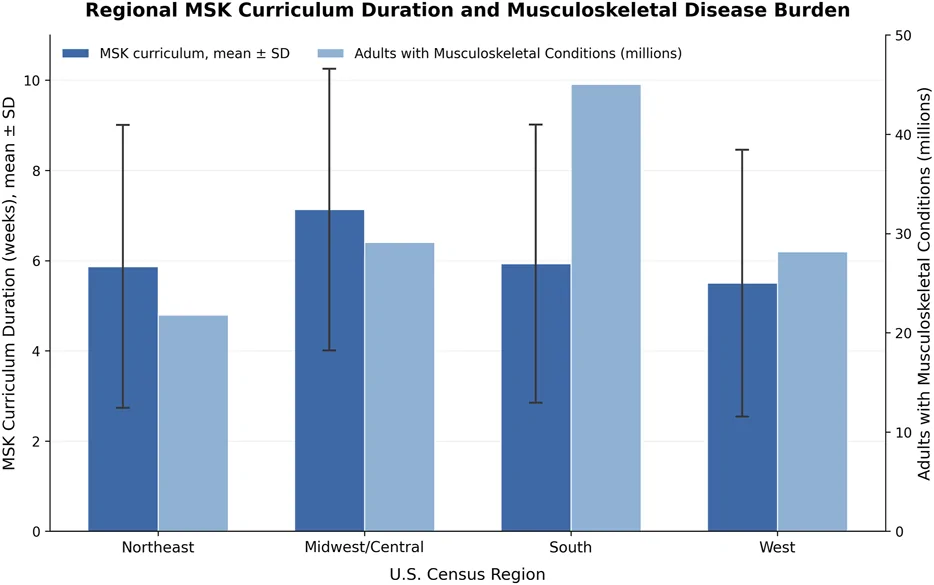
Regional ambulatory MSK visit frequency compared with mean MSK curriculum duration across U.S. medical schools. Regions with higher outpatient MSK visit volumes, including the Midwest and South, did not demonstrate increased MSK curriculum duration. Mean curriculum duration remained relatively consistent across regions. MSK = musculoskeletal.

**Fig. 2:**
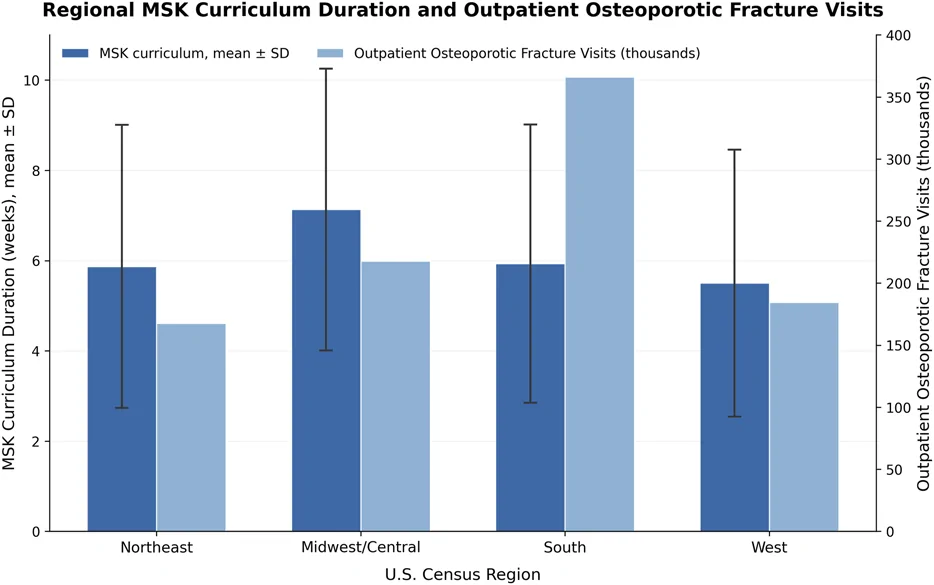
Regional osteoporotic fracture–related visit frequency compared with mean MSK curriculum duration. Despite higher fracture-related healthcare utilization in the Midwest and South, MSK curriculum duration remained similar across all geographic regions. MSK = musculoskeletal.

**Fig. 3:**
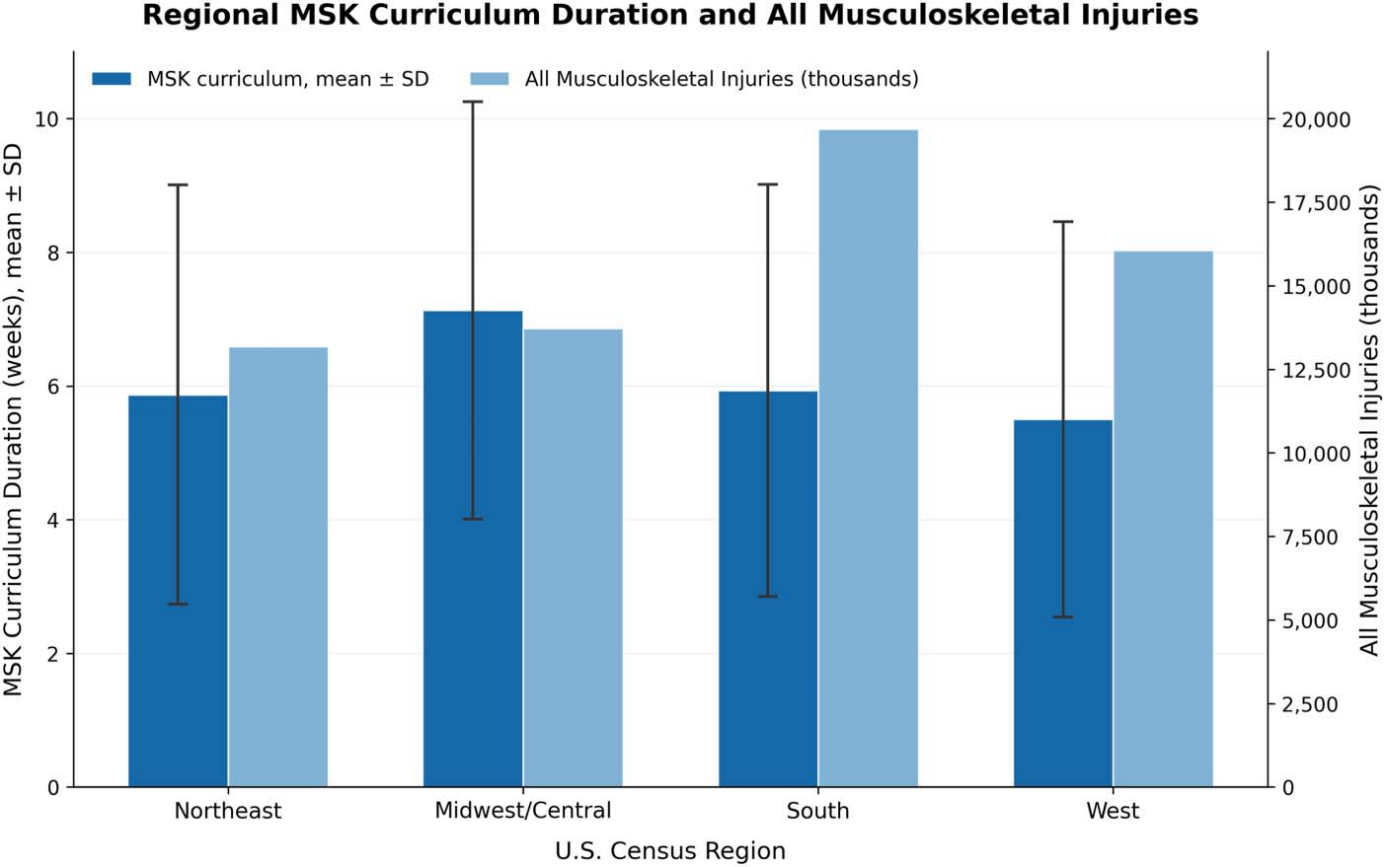
Regional acute musculoskeletal injury frequency compared with mean MSK curriculum duration. Acute MSK injury rates were consistently high across all regions; however, curriculum duration demonstrated minimal variability, suggesting no alignment between injury burden and educational exposure. MSK = musculoskeletal.

## Discussion

We selected 3 BMUS indicators that represent common musculoskeletal conditions encountered across primary care and orthopaedic practice. Although these measures do not capture the full spectrum of musculoskeletal disease, they provide standardized national estimates of population burden and therefore offer a practical framework for evaluating broad relationships between regional disease burden and curriculum duration.

In this national study of 78 of 157 U.S. allopathic medical schools (50%) with publicly available MSK curriculum data, we found that average MSK instructional duration was approximately 6 weeks and did not vary significantly by institutional mission or regional disease burden. Our findings demonstrate that U.S. allopathic medical schools devote limited curricular time to musculoskeletal education, despite substantial regional variation in musculoskeletal disease burden across the United States^5,6^. Notably, MSK curriculum duration does not differ significantly by primary-care mission, geographic region, or local MSK disease burden^15^. These results highlight a consistent and systemic underemphasis on MSK training despite the high prevalence of MSK disorders and their central role in clinical practice.

MSK disorders affect more than 30% of U.S. adults and account for more than 20 million ambulatory visits per geographic region annually^1^. Back pain alone is among the most common reasons for seeking medical care in the United States^1^. Previous literature has consistently described musculoskeletal education as comprising a relatively small portion of undergraduate medical education^5,6^. However, because comprehensive comparisons with instructional time devoted to other specialties have not been performed, this characterization should be interpreted within the context of the existing musculoskeletal education literature rather than as a direct comparison across disciplines. This finding is particularly concerning given that MSK complaints constitute up to 40% to 60% of primary care visits, and primary care providers are often the first point of contact for patients with MSK conditions^1^.

When primary care clinicians lack sufficient musculoskeletal training, referral rates to orthopaedic specialists increase, contributing to longer wait times and higher healthcare expenditures^1^. Strengthening MSK training within primary care oriented curricula may therefore represent an important strategy for improving healthcare system efficiency.

A key goal of this study was to determine whether medical schools in regions with disproportionately high MSK disease burden appropriately increased MSK training. According to our findings, MSK curriculum duration did not differ significantly across the 4 major AAMC regions, nor was there a difference when analyzed at the more granular 9-region level. For example, the South and Midwest/Central regions exhibit substantially higher burdens of arthritis, osteoporotic fractures, and chronic joint pain, yet medical schools in these regions offer MSK instruction of similar duration to schools in regions with lower disease burden. This disconnect suggests that curriculum is largely independent of local epidemiology and may be more heavily influenced by historical patterns, predetermined curricular structures, or limited instructional resources. The key contribution of this study is demonstrating that current MSK curriculum structures appear largely fixed across institutions rather than reflecting regional epidemiologic needs.

We recognize that regional disease burden is only 1 of many factors that may influence curriculum design. Likewise, medical school graduates do not universally remain in the regions where they complete their undergraduate education. However, many medical schools explicitly identify service to their surrounding communities as part of their institutional mission, and prior studies have demonstrated that physicians are more likely to establish practice near where they complete medical school or residency training^16-18^. Consequently, although regional epidemiology should not be viewed as the sole determinant of curricular content, it represents 1 reasonable consideration when evaluating how undergraduate medical education aligns with local healthcare needs. Future studies examining graduate practice patterns alongside curriculum characteristics may further clarify this relationship.

The implications of this misalignment are particularly pronounced in rural regions within high-burden areas where orthopaedic specialty access is limited. In these settings, primary care clinicians must manage a greater proportion of MSK disease^1,19^. Without adequate training, physicians may be less equipped to diagnose and manage MSK conditions efficiently, potentially delaying access to appropriate care, increasing referral rates, and elevating healthcare costs^20^. This challenge is compounded by the lack of standardized MSK competency assessments in undergraduate medical education, which limits accountability for educational outcomes.

The findings also show that primary-care emphasis was not associated with increased MSK curriculum duration. Given that MSK conditions are ubiquitous within the primary-care setting, this lack of curriculum expansion may reflect a continued underrecognition of the importance of MSK medicine rather than deliberate deprioritization. It may also reflect fragmentation of MSK teaching across anatomy, rheumatology, sports medicine, and orthopaedics, which may prevent centralized or expanded educational planning. These findings underscore the need for integrated MSK curriculum design that is reflective of the disease burden of the region and national expectations for competency assessment^20-23^.

An important structural barrier to expanding MSK education is the fixed duration of the medical school curriculum. Because MSK topics are typically embedded within broader surgery or medicine courses, increasing MSK instructional time would require reallocation from other disciplines^1,5,9,10,24,25^. This structural constraint may partially explain why MSK education has remained limited despite longstanding recognition of the problem.

International comparisons reveal similar challenges. In the United Kingdom, only 2.65 weeks of mandatory MSK education is provided, and fewer than half of graduating medical students report confidence in their MSK knowledge^25-27^. In India, fewer than 20% of students pass standardized MSK competency assessments, and Canadian medical schools face similar competency challenges^1,7,10,27^. These data collectively suggest that inadequate MSK education is a global phenomenon, likely driven by the absence of standardized competency benchmarks and insufficient prioritization of MSK care delivery^28^. Although this study does not identify the factors that determine curriculum design, these findings may help inform future discussions regarding how undergraduate musculoskeletal education can be evaluated alongside institutional priorities and broader educational goals.

This study has several limitations. MSK curriculum duration was derived from publicly available curricular information, which may incompletely capture integrated or longitudinal MSK instruction embedded within other courses. As a result, true educational exposure may be underestimated or variably reported across institutions. In addition, only U.S. allopathic medical schools with identifiable MSK curriculum data were included, which may limit generalizability. Regional MSK disease burden was assessed at the population level and may not fully reflect local patient populations served by individual institutions. Finally, curriculum duration was used as a surrogate for educational emphasis and does not account for instructional quality, teaching methods, or competency outcomes.

The cumulative evidence from this analysis and the existing literature indicates that MSK education is not structured in proportion to disease burden at the population level. Without improvements to MSK training, medical graduates may remain underprepared for providing competent primary care MSK management and supplying a robust pipeline of well-trained orthopaedic specialists. Establishing national curriculum standards, expanding MSK clerkships, and developing competency based assessments may be necessary steps toward aligning medical education with public-health needs.

## Conclusion

Musculoskeletal curriculum duration in U.S. medical schools appears largely independent of regional disease burden and institutional mission. This uniformity, despite high and variable population as well as demographic needs, highlights a fundamental misalignment between public health burden and educational emphasis at the medical school level. Addressing these gaps will require national curricular standards, competency-based benchmarks, and greater collaboration between undergraduate programs, residency training sites, and public health stakeholders. Without such reforms, future physicians may remain underprepared to manage the most common causes of disability and healthcare utilization in the United States.
